# A case report on mother-to-child transmission of Brucella in human, China

**DOI:** 10.1186/s12879-019-4302-y

**Published:** 2019-07-27

**Authors:** Guozhong Tian, Zhifei Zhan, Aimin Zhang, Hongyan Zhao, Xin Xia, Zixiang He, Bing Zhang, Menghua Zhao, Dongri Piao, Dianying Lu, Hai Jiang

**Affiliations:** 1 State Key Laboratory for Infectious Disease Prevention and Control, Collaborative Innovation Center for Diagnosis and Treatment of Infectious Diseases National Institute for Communicable Disease Control and Prevention, Chinese Center for Disease Control and Prevention, Beijing, China; 2 Hunan provincial Center for Disease Control and Prevention, Changsha, China; 3 Hunan People’s Hospital, Changsha, China

**Keywords:** Mother-to-child transmission, Brucella, China

## Abstract

**Background:**

Human brucellosis is endemic in China and commonly occurs through contact with infected animals from working with livestock or consumption of unpasteurized dairy products. Although rare, human-to-human, and possible sexual transmission, of Brucella has been reported. In this report, we describe a case of likely mother-to-child transmission of Brucella in Hunan Province, China.

**Case presentation:**

Between June and October 2016, a 28-year old man sought care for testicular swelling and pain at several health facilities. His 26-year old wife developed intermittent fever along with right thigh and hip pain between November 2016 and February 2017 respectively. On April 5, 2017, the female patient delivered a male neonate at 34 weeks of gestation through natural labor. The child’s venal blood sample was cultured on April 5, 2017. Brucella was isolated and identified on April 12, 2017. On the same date, serum antibodies of the father and mother were above 1:100 (based on the serum agglutination test [SAT]). The strains isolated from the mother and neonate were identified as *Brucella melitensis* biotype 1.

**Conclusions:**

This report highlights a family cluster of brucellosis. Culture results strongly support mother-to-child transmission, and a high probability of sexual transmission from husband to wife.

## Background

Human brucellosis remains an important zoonotic infection in China and is endemic in several provinces including Inner Mongolia, Shanxi, Heilongjiang, Hebei and Xinjiang [1]. Predominant strains include *B. melitensis* bv 3, *B. abortus* bv 1 and 3, *B. suis* bv 1 and 3 [1]. Human Brucellosis can range from asymptomatic infections to severe symptoms with fever, fatigue, loss of appetite, and joint-muscle and back pain. Brucella orchitis is reported among approximately 7% of male patients with brucellosis [2]. Symptom onset may occur between 5 days to 5 months following infection and may disappear and return several weeks or months later [3]. A suspect case of brucellosis is defined as a person with clinical symptoms and epidemiologic risk factors for infection. The serum (tube) agglutination test (SAT) is used to detect Brucella antibodies in China. Suspect cases with serum titers 1:100 and above are considered laboratory confirmed [4]. Culture and isolation can be performed at a few provincial-level laboratories and the national Brucella laboratory in Beijing.

Persons with suspect and laboratory confirmed Brucellosis are treated with Rifampin, Doxycycline or Streptomycin for 21 days [5]. Inappropriate treatment, poor adherence to treatment, or late diagnosis can lead to chronic infections. Chronic infections involve complex treatment and can last for several years [5]. Although transmission is commonly associated with contact with livestock (cattle, sheep, goats, and swine) and consumption of unpasteurized dairy products, sexual transmission has been previously reported [6–9]. In this report, we describe a case of likely mother-to-child transmission of Brucella in Hunan Province, China.

### Case presentation

In June 2016, a 28-year old male sought health care at People’s Hospital in Anhua County, Hunan Province for testicular swelling and pain. An ultrasound scan was performed and determined to be inconclusive; no treatment was provided. The patient subsequently developed a fever and received outpatient care at a local private clinic. In October 2016, the patient experienced recurrent swelling of the right testicle and again sought hospital care. He was treated for 7 days with Ciprofloxacin and Rifampicin. The patient improved and was discharged without a diagnosis.

In November 2016, a 26-year female, the wife of the 28-year old male patient and X months pregnant, developed a fever without obvious cause. She was diagnosed with acute nephritis at a local hospital, and, based on her complete blood count (CBC), was treated with Penicillin and Ampicillin for 3 days. In December 2016, the female patient sought care for a recurring fever and right thigh and hip pain. She was again diagnosed with acute nephritis and treated with Penicillin and Ampicillin for 15 days. In February 2017, the patient reported additionally unexplained right thigh and buttock pain and improved following analgesic therapy at home for 3 days.

**Fig. 1:**
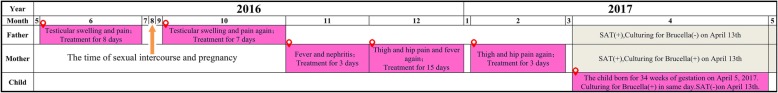
The clinical events on a family aggregation cases of brucellosis

On 5 April, 2017, the patient delivered a male neonate at 34 weeks of gestation through natural labor at People’s Hospital in Anhua County. At birth the neonate weighed 2500 g, with a length of 50 cm, but was in poor health (Apgar = 4) with a temperature of 37.6 °C along with an enlarged liver and spleen. The neonate was initially treated with a six-day course of Penicillin and Ampicillin. The clinical events of this family cluster of brucellosis are shown in Fig. 1.

### Laboratory diagnostics

The blood sample collected at the time of the neonate’s birth on 7 April was culture positive for Brucella on 12 April 2017 at People’s Hospital in Anhua County. Follow-up testing of blood samples from the father, mother, and neonate were also culture positive for Brucella on 12 April 2017 at Hunan People’s Hospital, Hunan Province. SAT results indicated that the father and mother had serum antibody titers above 1:100. The neonate’s white blood cell (WBC) count, platelet count and high sensitivity C reactive protein were higher than the normal; no serum antibody was detected. The strains isolated from the mother and the neonate were identified as *Brucella melitensis* biotype 1 and belonged to same genotype by MLVA genotyping (multi-locus variable-number tandem repeat analysis). The neonate was treated with Rifampicin and Doxycycline for one month at Hunan People’s Hospital. His parents were treated with Rifampicin and Doxycycline for a one-month period. All three patients - the father, mother, and neonate - were symptom-free at the end of the treatment period.

### Retrospective epidemiological survey

The family resided in urban housing, and the father’s worked in shipment of fruit from local farmers to urban markets, requiring travel throughout the province. The mother was not exposed to live livestock during pregnancy. The family regularly visited local markets for purchasing meat and dairy products for cooking; all identified dairy products were pasteurized. According to human surveillance data for the county, the risk of brucellosis was low and limited to *B. melitensis* biotype 3 from Chinese surveillance*.*

On April 18, 2017, an investigation was conducted for five close contacts of the family cluster of brucellosis. All contacts were asymptomatic and blood samples were culture and SAT negative for brucella and serum antibodies, respectively (Table 1).

**Table 1:** Epidemiological survey of close contacts with patients

Contacts	Sex	Age	Address	Occupation	Sampling time	Outcome	Relationship with patient	Comments
1	Male	53	Village	Farmer	April 18th	SAT - Culture -	Husband’s father	Lives with the patients
2	Female	50	Village	Farmer	April 18th	SAT -Culture -	Husband’s mother	Lives with the patients
3	Male	49	Town	Farmer	April 18th	SAT -Culture -	Wife’s father	Meals with the patients
4	Female	31	Town	Merchant	April 18th	SAT - Culture -	Wife’s sister	Meals with the patients
5	Male	4	Village	Student	April 18th	SAT - Culture -	Wife’s son	Lives with the patients

## Discussion and conclusions

The male patient described in this case report developed symptoms in June 2016. The patient was inadequately treated until receiving a Brucella diagnosis on 12 April 2017 following confirmatory SAT results. The patient’s wife and neonate were culture positive for *Brucella melitensis* biotype 1 and belonged to same genotype, suggesting mother-to-child transmission. The three patients were diagnosed as a family cluster of brucellosis.

Neither the male or female patient had previous travel to a brucellosis epidemic region, nor reported contact with sheep or other livestock. The family often consumed dairy products, including milk; however, no other cases were linked to the same these diary products. Although the male patient presented with typical symptoms at his initial contact with the health care system, the late diagnosis and inappropriate treatment led to a chronic brucella infection. The wife became pregnant in August 2016, a time between the male patient’s two disease episodes in June 2016 and November 2016. Because no other risk factors were identified, the wife was probably infected with Brucella through sexual transmission. Brucellosis transmission has occurred following blood transfusions or bone-marrow transplants [10]. Although uncommon, brucellosis has also been transmitted from suspicious person-to-person [11, 12].

The female patient developed clinical symptoms during pregnancy but was not successfully treated until after the birth of her son. Brucellosis causes fewer spontaneous abortions in humans than animals, primarily due to the absence of erythritol in the human placenta and fetus. The presence of anti-brucella activity in human amniotic fluid may also play a role. The MLVA-16 assays, which can differentiate closely related brucella isolates [1], revealed that the strains from the mother and her neonate had same genotype - B. melitensis biotype 1 – which is rare in China and the first detection of this strain in Hunan Province, a low risk are for brucellosis. Since the infant did not have any other possible exposures, these findings strongly support mother-to-child transmission of Brucellosis. The neonate’s negative test results for brucella antibodies may suggest an immature system unable to mount a detectable immune response from our previous test results.

Our report describes likely transmission of brucella from mother-to-child and probable sexual transmission from father to mother. Although these events are rare, this report highlights the importance of improving clinical awareness. Brucellosis should be considered for persons presenting with fever, fatigue, and joint-muscle pain not associated with other infections. Early and appropriate treatment is essential. With therapy during pregnancy, the overall success rate resulting in normal delivery is 90% [13].

## Data Availability

All original (de-identified) data and materials are available upon request from the corresponding author.

## Abbreviations

CBC: Complete Blood Count
MLVA: Multiple locus variable numbers of tandem repeats analysis
SAT: Serum agglutination test
